# Structural heart disease review of TAVR in low-risk patients: importance of lifetime management

**DOI:** 10.3389/fcvm.2024.1362791

**Published:** 2024-03-01

**Authors:** Mohamad B. Moumneh, Abdulla A. Damluji, Andras W. Heslop, Matthew W. Sherwood

**Affiliations:** Inova Center of Outcomes Research, Inova Heart and Vascular Institute, Fairfax, VA, United States

**Keywords:** TAVR, structural heart disease, aortic valve replacement, aortic stenosis, percutaneous coronary intervention

## Introduction

Aortic stenosis (AS) is the most prevalent form of valvular heart disease (VA), affecting millions of patients on an annual basis. It carries significant risk for morbidity and mortality if left untreated (1). Currently, no pharmaceutical therapy with proven effect in slowing the progression of AS exists. Thus, effective therapy relies on the timely diagnosis, surveillance in the early stages, and prompt treatment when indicated (2). Transcatheter aortic valve replacement (TAVR) has changed the landscape for treatment of AS, resulting in greater awareness and diagnosis of the disease, and prompting calls for earlier therapy. In addition, while TAVR was initially approved in high-risk symptomatic patients with severe AS or those with prohibitive risk who were poor candidates for surgery, it is now the preferred therapy for all risk levels and for most patients with AS (3). In this review, we aim to highlight the evidence supporting TAVR in low-risk patients, the guidelines for clinical use, and the continuing challenges for treatment of AS patients at both lower risk and younger ages.

## TAVR in low-risk patients – guidelines and clinical trials

Guidelines for the treatment of AS are derived from clinical trial evidence and currently implemented using the multidisciplinary “Heart Team” approach (4). The current American College of Cardiology/American Heart Association (ACC/AHA) guidelines recommend that all patients with severe AS undergo evaluation by a multidisciplinary team prior to consideration of percutaneous or surgical intervention (5). This includes imaging with echocardiography and/or more advanced imaging techniques, exercise treadmill testing when necessary, and consultation with both a cardiothoracic surgeon and a cardiologist with experience in TAVR (5). The guidelines have recently moved away from surgical/procedural risk stratification and toward a lifetime management approach in which the multidisciplinary team must consider the patient's age at the time of intervention, as well as their possible life expectancy, vascular and valvular anatomy, and symptoms. For patients with a poor vascular anatomy, prohibitive access, or for those with a valvular anatomy that is unfavorable for TAVR, surgical aortic valve replacement (SAVR) should be performed. For symptomatic patients aged >80 years with favorable anatomy, their life expectancy is likely ≤10 years, making TAVR the preferred choice over surgery. If these patients have a life expectancy of <2 years, shared decision-making is performed, and palliative or conservative options are considered (6). For symptomatic patients with a favorable anatomy and who are <65 years of age, life expectancy is often >10 years, and per the guidelines, SAVR is preferred. The balance comes for the symptomatic patients with favorable anatomies, between the ages of 65 and 80 years, where shared decision-making is recommended to make an individualized decision between TAVR and SAVR. Any patients with >20 years of life expectancy should be recommended for SAVR (5). These guidelines are based on the tremendous evidence base of randomized clinical trials (RCT) performed for TAVR vs. SAVR in patients at all risk levels. We will briefly highlight the PARTNER 3 and Evolut Low-risk trials in a later section of our review.

### Key takeaways

1. *Based on current guidelines, TAVR is recommended in symptomatic patients aged more than 80 years who have* ≤*10 years of life expectancy and a favorable anatomy*.
2. *SAVR is recommended in patients who either have a poor vascular anatomy, prohibitive access, unfavorable vascular anatomy for TAVR, >20 years of life expectancy, or are <65 years, symptomatic, and have a favorable anatomy*.
3. *Shared decision-making on the approach between SAVR and TAVR is recommended in symptomatic patients with a favorable anatomy aged between 65 and 80 years*.

## Durability of TAVR valves

When making decisions for a lifetime management of AS, especially on the use of TAVR in younger patients, the durability of the valve prosthesis is a critical factor in the decision-making process. Data on TAVR valve durability are limited because of the rapid pace of innovation and design of the early trials. There is a small amount of long-term data on the older valve generations, and more robust short-term data on the current generation of TAVR valves. The long-term data are from the NOTION trial, a multicenter RCT of 255 low-risk patients to either have SAVR or TAVR (7). The patients had a mean age of 79 years and were randomized to receive surgical bioprosthetic valves or the Medtronic CoreValve Classic. At 8 years of follow up, mortality rates for TAVR and SAVR patients were similar (51.8% vs. 52.6%; *p*-value = 0.90) (7). Valve performance showed some advantages for TAVR over SAVR with significantly lower rates of structural valve deterioration (13.9% vs. 28.3%; *p*-value = 0.0017) defined as a mean transvalvular gradient ≥20 mmHg, increase in mean gradient ≥10 mmHg from 3 months following the procedure, or new or worsening moderate intra-prosthetic aortic regurgitation from 3 months post the procedure; however, rates of bioprosthetic valve failure were similar between the two groups (8.7% vs. 10.5%; *p*-value = 0.61) defined as one of the following three criteria: (i) valve-related death; (ii) severe hemodynamic structural valve deterioration; and (iii) aortic valve reintervention. This was an important milestone for TAVR durability (7).

The recent data on current generation valves have utilized surrogate, echo-based outcomes. Forrest and colleagues evaluated the echocardiographic and clinical outcomes in the Evolut Low-Risk trial at 3 years of follow up (8). Among 1,414 patients, randomized to TAVR with CoreValve or SAVR, patients who underwent TAVR had significantly improved valve hemodynamics compared with patients who underwent SAVR (mean aortic valve gradient 9.1 vs. 12.1 mmHg; *p*-value <0.01), with no differences in all-cause mortality or disabling stroke (8). Early data from the PARTNER 3 trial were also favorable. In this study, 1,000 low-risk patients, with a mean age of 73 years, were randomized to receive either TAVR with the balloon-expandable SAPIEN 3 or SAVR. The primary composite endpoint of death, stroke, and rehospitalization at 1 year of follow-up was significantly lower in patients who underwent TAVR when compared with the surgical group (8.5% vs. 15.1%, 95% confidence interval, −10.8 to −2.5, *p*-value <0.001). These differences were sustained after 2 years in 96.5% of the patients who were available for follow-up although the rates of the primary composite endpoint were not statistically different after 5 years of follow up (9). Similarly, patients in the TAVR group at 30 days had lower rates of stroke (*p*-value = 0.02), death or stroke (*p*-value = 0.01), and new onset atrial fibrillation (*p*-value <0.001) (9). Such outcomes were not due to valve failure or deterioration, nor due to paravalvular leak following echocardiographic analysis on follow-up, indicating the early durability of TAVR valves (10). Based on the results of these two trials, the US Food and Drug Administration (FDA) approved the use of TAVR in low-risk patients in 2019 (https://www.fda.gov/news-events/press-announcements/fda-expands-indication-several-transcatheter-heart-valves-patients-low-risk-death-or-major).

Influenced by these trials, Tam et al. evaluated the durability of TAVR vs. SAVR by creating hypothetical cases in low-risk patients with severe AS using discrete event simulation (11). In their analysis, where mean age was 73.4 ± 5.9 years, the difference between SAVR and TAVR in terms of life expectancy was similar. In their modeling, TAVR durability would have to be 70% lesser than that of SAVR to change life expectancy, which they deemed a very unlikely scenario. They concluded that durability concerns should not affect the choice of TAVR vs. SAVR in all but the youngest (<60) low-risk patients (11).

**Figure 1:**
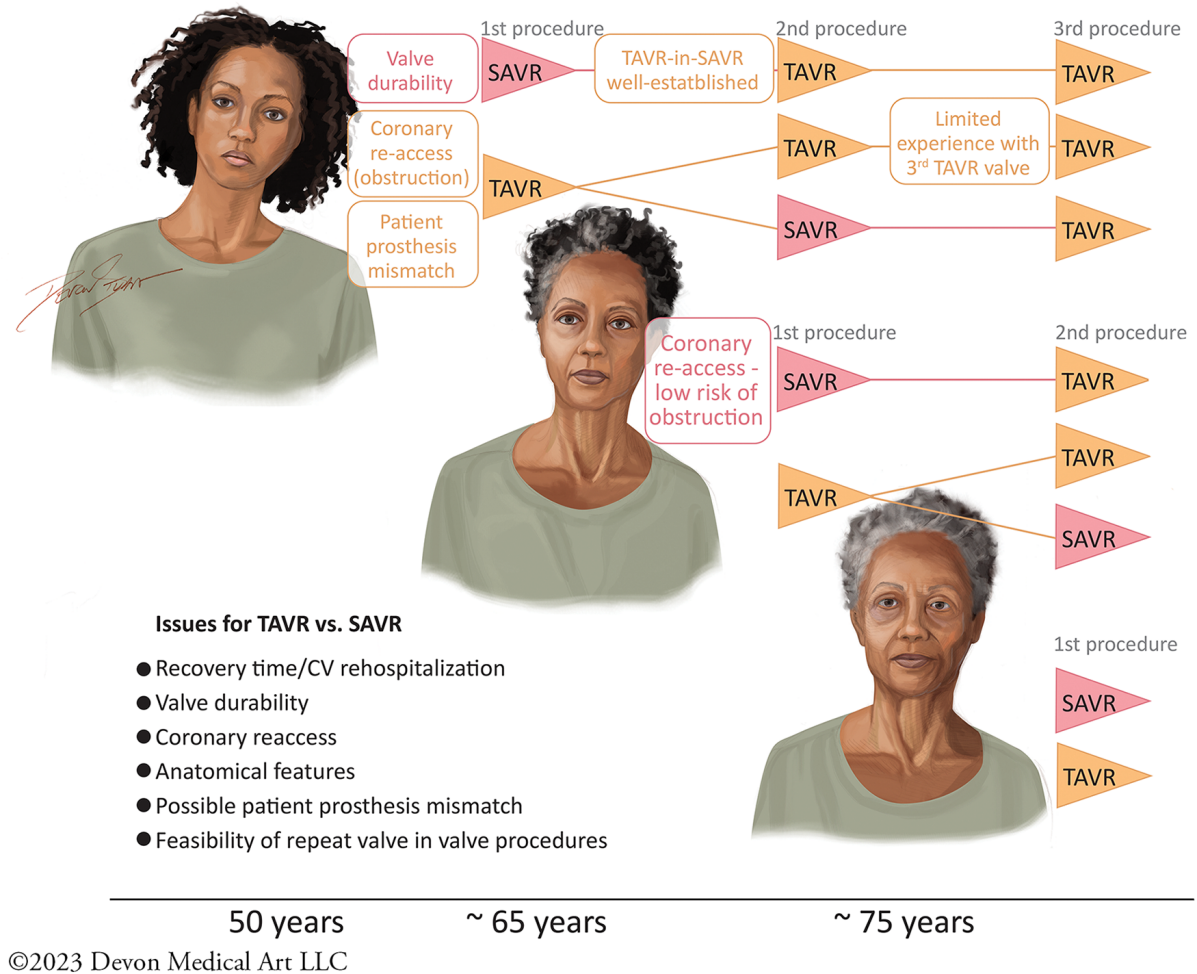
The therapeutic decisions for TAVR vs. SAVR in the treatment of AS are affected by multiple issues including questions of durability, recovery time, rates of rehospitalization, anatomical features, coronary re-access, creating patient prosthesis mismatch, and the feasibility of subsequent valve-in-valve procedures. These issues are dynamic, depending on the age of the patient considering therapy. I, Devon Stuart, Devon Medical Art, LLC, as the creator and copyright owner of the original work, “First AVR,” can confirm that the authors of this manuscript have been granted a limited license to authorize its reproduction in Frontiers in Cardiovascular Medicine. Owner website: devonmedicalart.com Owner email: devonmedicalart@gmail.com.

However, there are several factors, in addition to durability, that affect the choice of TAVR vs. SAVR (Figure 1). These include anatomical considerations like coronary re-access, and consideration for future valve interventions (depending on age) and risks. When considering coronary obstruction risk, the choice of SAVR valve may increase the risk in future valve interventions by four- to sixfold compared with TAVR, which impacts patients’ prognosis (12). The use of “unfriendly” surgical bioprosthesis including the stentless valves (i.e., Sorin Freedom and St. Jude Toronto) or internally stented valves (i.e., Sorin Mitroflow and St. Jude Trifecta), whose leaflets spread externally beyond the device frame, also increase the risk of coronary occlusion and they may best be avoided at the time of the first intervention (13, 14). These considerations warrant thoughtful deliberation when making a therapeutic decision at the outset of the first valve procedure.

### Key takeaways

1. *Limited data pertaining to valve durability exist, with most of the long-term data coming from the NOTION trial that shows favorable outcomes in TAVR over SAVR in terms of valve deterioration*.
2. *Trials like the Evolut Low-Risk, that showed better valve hemodynamics in TAVR, and the PARTNER 3, that showed a better primary composite endpoint of death, stroke, and rehospitalization in TAVR, led to FDA approval of TAVR use in low-risk patients*.

## Reintervention after TAVR

Most bioprosthetic valves, whether placed percutaneously or by conventional surgical techniques, eventually fail. Current estimates for surgical bioprosthetic valve durability are 10–15 years, though there is variation depending on valve type and patient factors (15). Methods of reintervention, for patients in need, have evolved since the introduction of TAVR. Valve-in-Valve TAVR for surgical bioprosthetic valves is an established procedure with known risks such as higher rates of coronary obstruction and possible patient prosthesis mismatch leaving the patient with a functional AS (16). TAVR-in-TAVR procedures are also performed at lower rates and present their own unique challenges. They introduce an increased likelihood of coronary obstruction, and thus preprocedural planning is important (17). Assessment of the original valve position with relation to the plane of the coronary ostia as well as the possible pinned leaflet plane for the valve prosthesis is important (18). Considering the mechanism of failure of the previous TAVR prosthesis is also important, as it may inform positioning of the next TAVR prosthesis and allow for consideration of risk vs. benefit with possible leaflet overhang. Finally, sizing of the previous TAVR is integral to TAVR-in-TAVR decisions as there is no likely benefit of the fracture of the previous prosthesis widening the valve orifice in these cases (19). In a study on 2,975 patients who underwent valve-in-valve TAVR with SAPIEN 3 and SAPIEN 3 ultra-valves, bioprosthetic valve fracture was used as an adjunct and was associated with higher risks of in-hospital mortality and bleeding, with little improvement in hemodynamic status on echocardiography (20). Continued research and advancing technology will make these procedures safer.

In a minority of cases, TAVR in TAVR is not feasible and TAVR explantation with redo SAVR is necessary. These procedures are more complex than conventional first-time SAVRs, and perhaps more morbid than conventional redo SAVRs. Limited data exist and are mostly from past generation devices. In terms of TAVR explantation risks, Bapat et al. evaluated the clinical and echocardiographic outcomes in the EXPLANT TAVR registry at 30 days and 1 year of follow-up. In this international multicenter registry on 269 patients with a median age of 72.7 years who underwent TAVR explantation, overall survival, in-hospital mortality, 30-day mortality, and 1-year mortality rates were 76.1%, 11.9%, 13.1%, and 28.5%, respectively. These results demonstrate that risks of TAVR explantation should not be negated but taken into consideration during AS management (21).

### Key takeaways

1. *When deciding on methods of reintervention such as TAVR in TAVR, several factors must be considered such as the position of the original valve with respect to the ostia, mechanism of failure of the previous TAVR, and sizing of the previous TAVR*.
2. *When TAVR in TAVR is not feasible, TAVR explantation is required; however, risks should be evaluated as indicated by the EXPLANT TAVR registry*.

## Coronary access after TAVR

As the number of TAVRs in young patients with severe AS is increasing, an increase in the number of patients receiving coronary angiography or percutaneous coronary intervention (PCI) following TAVR is anticipated (22). Preservation of coronary access (CA) is thus pivotal. CA following TAVR is a rigorous task mainly influenced by the interaction between the coronary ostia and the transcatheter heart valves (THV). Orientation of these THVs is crucial in determining success rates of coronary cannulation and access. While supra-annular THVs like the Evolut R/Pro and Acurate Neo provide mechanisms to improve commissural alignment, intra-annular THVs like the SAPIEN 3 do not. However, the SAPIEN 3 valve is unique in that its shorter frame height often allows for unobstructed access to the coronaries, possibly reducing the need for commissural alignment in select cases. Complications in CA are observed when two THVs come into play. One THV might push the outflow of the first, that is close to or at the sinotubular junction, resulting in blockage of the blood flow toward the aortic sinus (23).

Evaluating CA following TAVR is often conducted by coronary angiography or computed tomography (CT); however, data pertaining to the aforementioned modalities remain limited. In the study by Ochiai et al., 66 patients were treated with the Evolut R/Pro THVs, while 345 were treated with the SAPIEN 3 THVs. Unfavorable CA on CT was observed in 34.8% and 25.8% for the left coronary and right coronary arteries, respectively, with Evolut R/PRO, compared with 15.7% and 8.1% in patients treated with SAPIEN 3. The study also showed superior success rates in terms of coronary engagement in patients with favorable CA in all types of THVs (24). As for studies involving coronary angiography, the ALIGN-ACCESS was the first to address this. Its results indicated that commissural alignment increases the rate of selective coronary cannulation after TAVR with THVs like the Evolut R/Pro and Acurate Neo, despite having a higher risk of impaired CA when compared with the SAPIEN 3. In addition, the study showed that patients with a misaligned Evolut R/Pro and Acurate Neo, lower sinus of Valsalva (SoV), and higher THV to SoV relation are at a greater risk of impaired CA after TAVR (25). In the single-center prospective RE-ACCESS study, 7.7% of patients had unsuccessful coronary cannulation following TAVR, with 96% of those cases reported with the Evolut R/Pro THVs. Similar to the ALIGN-ACCESS, this study showed that a higher THV to SoV relation was a major predictor for increased risk of unsuccessful coronary cannulation following TAVR. Another predictor was decreased depth of implantation (26). An additional anatomical feature that might influence CA includes whether the aortic valve is bicuspid or tricuspid. Scarce data comparing CA following TAVR in bicuspid and tricuspid valves exists. Bicuspid aortic valves (BAV) are often divided into subtypes based on Siever's anatomical classification: Type 0 indicating no raphe; Type 1 indicating a fused raphe (i.e., between the left and right cusps); Type 2 indicating two fused raphes. In one study, THVs were used in 86 type 0 BAV, 70 type 1 BAV, and 132 tricuspid aortic valves (TAV). The results indicated that type 0 BAV had fewer THV-related challenges in CA in the left coronary artery compared with the TAV, indicating favorable outcomes; however, additional studies and a larger sample population are needed to further verify this in the future (27). Another factor that should be considered in CA following TAVR is the stent frame. In the study by Kim et al. on 449 patients from 25 centers, short stent-frame prosthesis (SFP), defined as any balloon-expandable transcatheter heart valve, ACURATE platform, Centera, DirectFlow, Engager, JenaValve, and Lotus had significant rates of success of CA following TAVR when compared with longer SFP, defined as Evolut platform and Portico in the right coronary (99.6% vs. 95.9%, *p*-value = 0.005), but not in the left coronary artery (99.7% vs.98.7%, *p*-value = 0.24).

One can thus conclude that the major factors hindering CA following TAVR are either anatomical, like a narrow SoV or a low coronary ostial height, or device and procedure related, like depth of implantation, position of commissures, and length of frame. With the experience of physicians and screening techniques like CT, appropriate patient selection and avoidance of CA complications are feasible (28).

### Key takeaways

1. *CA following TAVR is often evaluated by CT or coronary angiography. Its preservation is of utmost importance*.
2. *As indicated by various trials, a multitude of anatomical and procedural factors affect CA following TAVR including ostial height, narrow SoV, length of frame, and depth of implantation*.

## Progression and treatment in moderate AS

While the comprehensive documentation of severe AS in symptomatic patients is well established, there seem to be less data related to the clinical impact of moderate AS compared with mild or no AS. Hence, it is critical to understand the progression of moderate AS by evaluating the anatomical and clinical aspects of its progression. Regarding its anatomical progression, hemodynamic AS, which is usually homogenous with an average increase in peak velocity of 0.3 m/s per year, varies between patients. In terms of its clinical progression, patients often have a benign prognosis and an average of 13.4 years before developing severe AS requiring surgery. However, recent data have indicated that moderate AS is associated with increased mortality rates due to both cardiac and non-cardiac causes (29). This was demonstrated in a large Australian registry of 3,315 patients, where moderate AS was associated with long-term mortality, and had mortality risks similar to those seen in patients with severe AS (5-year mortality: moderate AS 56% vs. severe AS 67%) (30). As for factors affecting progression of moderate AS, results from a multivariate analysis indicated that a thickened left ventricular posterior wall, renal impairment, and aortic valve area were significantly associated with an increase in the fast progression of moderate AS into severe AS when compared with a slower progression. Moreover, faster progression was independently associated with increased risk for all-cause mortality. Hence, faster progression of moderate AS resulted in worse overall outcomes (31).

In terms of management of moderate AS, guidelines fail to provide specific recommendations. In the Simvastatin and Ezetimibe in Aortic Stenosis (SEAS) trial, a randomized double-blind trial on 1,873 patients with mild to moderate AS who received either simvastatin plus ezetimibe or placebo daily, medical treatment did not show any improvements in preventing the progression of moderate AS or its adverse clinical outcomes (32). TAVR, as previously mentioned, is also not indicated in management and prevention of progression. Ongoing trials, like the TAVR UNLOAD in patients with moderate AS and heart failure (HF) with reduced left ventricular ejection fraction (LVEF) and the PROGRESS and Evolut EXPAND TAVR II Pivotal in patients with moderate AS and/or cardiac damage and dysfunction, might eventually show TAVR's role as a treatment option for moderate AS.

### Key takeaways

1. *Limited data and guidelines on the progression and treatment of moderate AS exist. Results from a certain registry and multivariate analysis indicate that the risk of mortality in moderate AS is similar to that of in severe AS, with factors such as thickened left ventricular posterior wall, renal impairment, and aortic valve area significantly influencing moderate AS progression*.
2. *Similarly, there is limited information about the role of TAVR in the treatment and prevention of progression of moderate AS. Ongoing trials might provide future insights about TAVR's role*.

## Asymptomatic patients with severe AS

The management of asymptomatic patients with severe AS is a challenging task, with persistent debates related to optimal timing and methods of intervention (SAVR vs. TAVR) existing among physicians. While some claim that patients with delayed intervention are prone to irreversible outcomes of HF or death, the European Society of Cardiology (ESC)/European Association for Cardio-Thoracic Surgery (EACTS) recommends the “watchful waiting” approach, as this category of patients has good prognosis (33). The approach also suggests that intervention should not be performed unless certain symptoms and clinical findings are present, such as severe left ventricular hypertrophy, a systolic pulmonary artery pressure of more than 60 mmHg, increases in pro-brain natriuretic peptide (BNP) by more than threefold its normal value on follow-up, the mean gradient increases by >20 mmHg with exercise, increasing size of the left atrium, and decreases in the indexed stroke volume, as well as decreases in the left ventricular global longitudinal strain by more than −14.7% (34).

As for guidelines related to asymptomatic severe AS, several possible indications for aortic valve replacement (AVR) exist. These include LVEF < 50%, low-surgical-risk patients with severe AS (mean gradient >60 mmHg, aortic velocity >5 m/s), or severe AS with BNP level >3 times its normal range, or severe AS with an increase in aortic velocity >0.3 m/s per year, or decreased exercise tolerance or a drop in systolic blood pressure (SBP) >20 mmHg from baseline to peak exercise during carefully supervised bike or treadmill testing (using cardiopulmonary exercise testing protocols or standard/modified BRUCE protocols) (35).

However, two RCTs, namely, Randomized Comparison of Early Surgery Versus Conventional Treatment in Very Severe Aortic Stenosis (RECOVERY) and Aortic Valve Replacement Versus Conservative Treatment in Asymptomatic Severe Aortic Stenosis (AVATAR), dispute the “watchful waiting” approach. The RECOVERY trial compared the safety and efficacy of early surgical intervention vs. watchful waiting in asymptomatic patients with severe AS. These studies did not use the aforementioned risk factors (BNP, LV-indexed stroke volume, LV strain, etc.) to identify patients for early intervention; instead they randomized asymptomatic patients with severe AS for continued surveillance for the development of symptoms vs. early surgical intervention. They reported superior outcomes in the early surgical intervention group in terms of cardiovascular mortality at 4 years (1% vs. 15%, *p*-value <0.05), all-cause mortality at 8 years (10% vs. 32%, *p*-value <0.05), and HF hospitalization (0% vs. 11%, *p*-value <0.05) (36). Similarly, the AVATAR trial, which compared asymptomatic patients with severe aortic stenosis undergoing AVR vs. conservative therapy, reported superior outcomes in the intervention group in terms of all-cause mortality, HF, acute myocardial infarction (AMI), and stroke (15.2% vs. 34.7%, *p*-value = 0.02) (37). It is important to note that certain limitations pertaining to these two studies exist. These include the small sample size and highly selected population, where the RECOVERY trial included patients having a valve area ≤0.75 cm^2^ with a peak velocity ≥4.5 m/s or mean gradient ≥50 mm Hg, and the AVATAR trial only included patients able to complete the exercise stress test, while excluding patients with chronic health conditions or high perioperative risks (33).

In terms of TAVR, limited information regarding its role in asymptomatic patients is known. A recent study, evaluating the role of TAVR in 231,285 patients with AS, reported superior survival rates after 1 year following TAVR in patients with minimal symptoms when compared with patients with moderate-to-severe symptoms (adjusted hazard risk for death: 0.70, 95% CI: 0.66–0.75). Similarly, the mean change in the Kansas City Cardiomyopathy Questionnaire Overall Summary Score (KCCQ-OS) in this study, which included variables such as physical limitation, social limitation, and quality of life scores, was lower in the minimal symptom group following TAVR (2.7 point and 3.8 points vs. 32.2 and 34.9 points) increases at 30 days and 1 year, respectively (38). Another RCT, the EARLY-TAVR, comparing asymptomatic patients receiving TAVR with the balloon-expandable SAPIEN 3 prosthetic vs. conservative therapy, has recently completed enrollment, and upon its primary completion in March 2024 is expected to provide future insight into the role of TAVR in asymptotic patients (39).

### Key takeaways

1. *Physicians often debate the optimal timing and methods of intervention when managing asymptomatic AS patients. Some encourage the watchful waiting approach that defers intervention unless certain symptoms such as severe left ventricular hypertrophy, increases in: levels of BNP/NT-proBNP, the mean gradient, and the size of the left atrium; and decreases in: the indexed stroke volume and left ventricular global longitudinal strain are present. However, trials like the RECOVERY and AVATAR dispute the watchful waiting approach*.
2. *There is scarce data on TAVR's role in asymptomatic patients with AS, with future and ongoing trials expected to expand knowledge on its role*.

## Conclusion

Multiple factors must be taken into consideration when deciding on a treatment approach between SAVR and TAVR in low-risk patients with AS. Such factors include patient preference and anatomical, procedural, and surgical expertise. Based on the results of certain trials, TAVR was shown to have a role in treating these patients. As a result, exploring the use of TAVR in low-risk patients is of paramount importance in the changing field of cardiovascular disorders. Not only does TAVR provide a current solution for low-risk patients with AS, but it also acts as a blueprint for the continuous comprehension and future management of these patients. With the constant challenges faced and the limited evidence about its role in this subcategory, it is crucial for ongoing as well as future trials to resume their work and research in an effort to enhance both quantity and quality of patients’ lives.
